# How autophagy, a potential therapeutic target, regulates intestinal inflammation

**DOI:** 10.3389/fimmu.2023.1087677

**Published:** 2023-04-24

**Authors:** Shuang-Lan Chen, Chun-Meng Li, Wei Li, Qing-Song Liu, Shuang-Yuan Hu, Mao-Yuan Zhao, Dong-Sen Hu, Yan-Wei Hao, Jin-Hao Zeng, Yi Zhang

**Affiliations:** ^1^ Department of Gastroenterology, Hospital of Chengdu University of Traditional Chinese Medicine, Chengdu, China; ^2^ Department of Reproductive Medicine, Chengdu Xinan Women’s Hospital, Chengdu, China; ^3^ TCM Regulating Metabolic Diseases Key Laboratory of Sichuan Province, Hospital of Chengdu University of Traditional Chinese Medicine, Chengdu, China

**Keywords:** signaling pathway, inflammatory bowel disease, autophagy, autophagy-associated gene, endoplasmic reticulum stress, intestinal microflora

## Abstract

Inflammatory bowel disease (IBD) is a group of disorders that cause chronic inflammation in the intestines, with the primary types including ulcerative colitis and Crohn’s disease. The link between autophagy, a catabolic mechanism in which cells clear protein aggregates and damaged organelles, and intestinal health has been widely studied. Experimental animal studies and human clinical studies have revealed that autophagy is pivotal for intestinal homeostasis maintenance, gut ecology regulation and other aspects. However, few articles have summarized and discussed the pathways by which autophagy improves or exacerbates IBD. Here, we review how autophagy alleviates IBD through the specific genes (e.g., *ATG16L1*, *IRGM*, *NOD2* and *LRRK2*), crosstalk of multiple phenotypes with autophagy (e.g., Interaction of autophagy with endoplasmic reticulum stress, intestinal antimicrobial defense and apoptosis) and autophagy-associated signaling pathways. Moreover, we briefly discuss the role of autophagy in colorectal cancer and current status of autophagy-based drug research for IBD. It should be emphasized that autophagy has cell-specific and environment-specific effects on the gut. One of the problems of IBD research is to understand how autophagy plays a role in intestinal tract under specific environmental factors. A better understanding of the mechanism of autophagy in the occurrence and progression of IBD will provide references for the development of therapeutic drugs and disease management for IBD in the future.

## 1 Introduction

The inflammatory bowel diseases (IBD) are described as complex, recurrent inflammatory conditions which are manifested as Crohn’s disease (CD) and ulcerative colitis (UC). The common clinical symptoms of IBD include severe diarrhea, abdominal pain, and weight loss, among others. If left untreated, the condition can lead to serious complications or the development of colorectal cancer (CRC). The pathogenesis of inflammatory bowel diseases (IBD) is multifactorial and complex, including an unfavorable environment, susceptibility gene variants, abnormal intestinal microbiota, and a mucosal immune and inflammatory response genetically susceptible to the host microbiota (1, 2), but its specific pathogenesis is still not fully elucidated.

Autophagy is a conserved intracellular degradation pathway that helps maintain intracellular homeostasis during stress or malnutrition. Autophagy is prevalent across cells and interacts with other essential cellular homeostatic processes. However, in the intestinal mucosa, cell-type-specific differences in autophagic function may exist (3). A growing number of studies suggest that autophagy may mediate the pathophysiological process of IBD. For example, autophagy regulates the clearance of invading pathogens. When bacteria infect host cells, cytoplasmic vesicles engulf the pathogens to form autophagic vesicles, thereby limiting the pathogens’ access to nutrients. Enhanced autophagy promotes the integration of autophagic vesicles and lysosomes for the degradation of pathogenic microorganisms (4–6). Autophagy maintains cell survival by protecting cells from bacterial toxins, such as intestinal epithelial cells (IECs) and macrophages (7). Autophagy also mediates the functions of innate and adaptive immunity, such as antigen delivery by dendritic cells, inflammatory factor secretion by macrophages, and antimicrobial peptide production by Paneth cells (8, 9). In contrast, autophagy inhibition leads to inflammation and increases the susceptibility to CD (10–12). In summary, impaired autophagy can cause intestinal cell dysfunction, dysbiosis of intestinal microbial and uncontrolled immune response, leading to intestinal inflammation (13–16).

Based on the important impact of autophagy on the gut, in this review, we will discuss how autophagy affects intestinal cells and gut function. We will also discuss how autophagy plays a role in the development of IBD *via* various pathways, including the genes, cell signaling pathways, ERS, and microorganisms involved. Meanwhile, a brief summary of the current research status of IBD therapeutic agents targeting autophagy is presented. These will further explain the pathogenesis of IBD and provide novel ideas for the development and use of drugs for IBD treatment in the future.

## 2 Autophagy and the underlying mechanism

In eukaryotic cells, autophagy is an important protein degradation system and a tightly regulated catabolic and organelle renewal process (17). In this process, the cells’ own cytoplasmic proteins and organelles are encapsulated into the vesicles. The vesicles then fuse with lysosomes to form autophagic lysosomes that degrade their encapsulated contents. There are three types of autophagy: macroautophagy, microautophagy, and chaperone-mediated autophagy (CMA) (18).

Macroautophagy is a process in which the endoplasmic reticulum, Golgi apparatus, or cytoplasmic membrane surround the material to be degraded into bimodal binding vesicles called autophagosomes. The autophagosome then fuse with lysosomes and degrade the contents. Microphagy implies that the lysosomal membrane directly wraps the long-lived proteins, among other components, and degrades them within the lysosome. CMA is a process of selective protein degradation through a lysosome-dependent mechanism. In this process, intracytoplasmic proteins bind to molecular chaperones and are translocated into the lysosomal compartment and subsequently digested by lysosomal enzymes (19).

**Figure 1:**
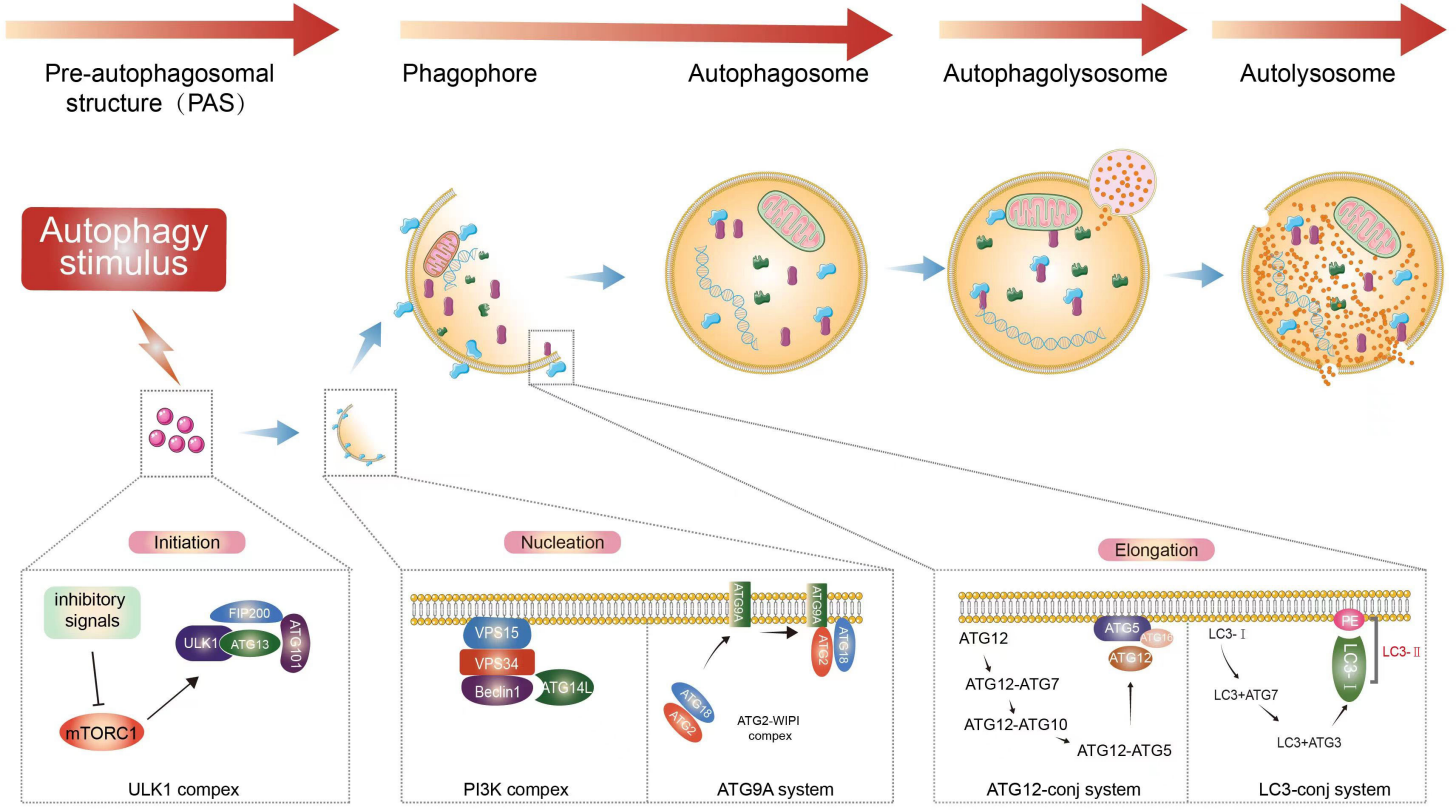
Autophagy process. Extracellular stimuli or recognition of substances that induce the formation of autophagosomes. Macroautophagy starts with inhibitory signals acting on mTOR (20). The ULK1 complex is an important upstream initiator that induces the activation of nucleation complexes such as PtdIns3K and BECN1, which in turn binds to autophagosomes and induces the prolongation of phagocytic membranes (21–23). Binding of LC3 to autophagosomes and control of autophagosome maturation and extension (24, 25). After vesicle formation, autophagosomes fuse with lysosomes to form autophagic lysosomes, releasing the contents (26–28). This is followed by the subsequent degradation of the contents occurs.

Macroautophagy can be roughly divided into four stages (**Figure 1**): initiation of autophagy, formation of isolation membranes and autophagosomes, fusion of autophagosomes with lysosomes, and cleavage of autophagosomes. Autophagy is initiated by the unc-51-like autophagy-activated kinase 1 (ULK1) complex, which phosphorylates its downstream autophagy proteins FIP200 and autophagy-associated gene 13 (ATG13) (21–23). The transmembrane binding of ATG9 provides the double membrane for the formation of phagosomes, primarily from the endoplasmic reticulum (ER), through the Beclin 1 (BECN1)-phosphatidylinositol-3-kinase (PtdIns3K) complex to produce phosphatidylinositol-3-phosphate (PtdIns3P), which then recruits the WIPI complex (ATG2-ATG18 in yeast). This is followed by phagocytic membrane elongation (24, 25). With the formation of the ATG12 conjugation system (comprising the ATG5-ATG12-ATG16L1 complex, which requires ATG7 and ATG10 activity to form), microtubule-associated protein 1 light chain 3 alpha (MAP1LC3A/LC3) binds to phosphatidylethanolamine (PE) *via* ATG7 and ATG3, leading to the maturation of autophagosomes (26–28).

## 3 Autophagy is closely associated with IBD

### 3.1 Absence or mutations in specific genes of autophagy pathway interfere in IBD

At the genetic level, the autophagy-related 16-like 1 gene (*ATG16L1*) and immunity-related GTP-ase family M gene (*IRGM*) as risk factors for CD brought autophagy into focus in IBD (29–32). Subsequent studies have shown that mutations in the autophagy-related genes ULK1, leucine-rich repeat kinase 2 gene (*LRRK2*), and nucleotide-binding and oligomerisation domain 2 gene (*NOD2*) are strongly associated with the development of IBD (33–35).

#### 3.1.1 ATG16L1

*ATG16L1* is a particularly important autophagy gene. In a German study, a genome-wide association scan of nonsynonymous SNPs identifies a susceptibility variant for Crohn disease in *ATG16L1* (29). However, studies in several Asian countries, including Japan, Korea and China, did not show an association between *ATG16L1* gene variants and CD (36–38). In the analytical study of correlation with phenotype, A*TG16L1* mutations were associated with intestinal luminal stricture and perianal infiltration, and the number of risk alleles was positively associated with the risk and severity of CD development (39).

Cysteine aspartate proteases (caspase) are a group of proteases present in the cytoplasm that are closely related to cell growth, differentiation and especially apoptosis. Thr 300-to-Ala (T300A) polymorphism in *ATG16L1* has been found to be a serve as a critical susceptibility factor for CD. It can promote caspase 3-dependent degradation during cellular stress, leading to protein instability (40). In a proapoptotic context, cleaved forms of caspase 3 are retransplanted to degrade BECN1, which hinders the initiation of autophagy (41). *ATG16L1* ^T300A^ expression is associated with an increased risk of CD in response to the reduction of bacterial capture rates and impaired bacterial processing through autophagy. The *ATG16L1* coding variant was found to be impaired in the capture of internalized *Salmonella* in autophagosomes in human IECs (42). Interleukin 22 (IL-22) directly acts on IECs and contributes to the intestinal immune response to pathogen infection (43, 44) and epithelial wound healing (45). However, under certain conditions, IL-22 may cause tissue damage (46). A recent study showed that IL-22 signaling in IEC is regulated by gene *ATG16L1* and drives intestinal inflammation in an autophagy-dependent manner (47). *ATG16L1* regulation of autophagy is also cell-specific. For example, under inflammatory conditions, *ATG16L1* deletion in myeloid cells exerts limited effect on IBD, whereas *ATG16L1* deletion in ICE will increases IEC apoptosis, aggravates chronic inflammation. However, IECs can be maintained without autophagy as long as intestinal homeostasis is maintained (48).

#### 3.1.2 IRGM

*IRGM* is thought to be a shared susceptibility gene for both CD and UC (49). However, it plays a more prominent role in the pathogenesis of CD, and its role in UC seems to be controversial. Because, data from several studies showed a strong association between *IRGM* variants and CD susceptibility (31, 50, 51), while there was no significant association between susceptibility to UC (50, 52, 53). Further, in a study of the effect of *IRGM* variants on IBD susceptibility, it was shown that the association signal of *IRGM* with CD was considerably weaker compared to *ATG16L1* (50, 54).

*IRGM* exerts resistance to intracellular infection through various mechanisms, including regulation of phagosome processing, cell motility, and autophagy. Protein IRGM has no homologs among the ATG genes in yeast, which makes it difficult to assign to it an autophagy-specific function; instead, IRGM has been considered to affect autophagy indirectly (55, 56). IRGM interacts with ULK1 and BECN1 and promotes their co-assembly, thereby regulating the formation of the autophagy initiation complex. Meanwhile, it physically pairs with autophagy protein NOD2 and ATG16L1 to exert antibacterial and anti-inflammatory effects (57). *IRGM* polymorphisms (rs13361189, rs4958847, and rs11741861) are considered to increase the susceptibility to CD and UC (51, 58). *IRGM1* expression deficiency significantly increases the susceptibility to chemically-induced colitis in part by improving the accessibility of commensal bacteria to intestinal tissues (59, 60). For example, altered *IRGM* expression are predicted to be associated with CD, affecting the autophagic control of S*almonella typhimurium* (32). The loss of tight *IRGM* expression regulation disrupts replication in CD-associated adherent-invasive *Escherichia coli* controlled through autophagy (61).

#### 3.1.3 NOD2

Genetic polymorphism in NOD2 was the first found to be associated with the risk of CD development and confers the strongest genetic risk (62–64). Around 40–50% of CD patients carry at least one mutation in the NOD2/CARD15 gene (65, 66). However it has heterogeneity and many studies have shown that NOD2 variants play no role in CD in East Asian populations (67–69). In a systematic review and meta-analysis on the association between identified NOD2 mutations and the prognosis of complex CD, it was shown that every NOD2 mutation was associated with a 58% increased risk of surgery (70)

The most common IBD-associated polymorphisms in NOD2 are the amino acid mutations R702W, G908R, and L1007fs. These mutations occur in the C-terminal leucine-rich repeat structural domain responsible for detecting cytoplasmic MDP and have been shown to cause defects in the perception of this bacterial-derived molecule (71, 72). Protein NOD2 directly intersects with autophagy and induces protein ATG16L1 to recruit bacteria to the entry site, where it triggers bacterial autophagy (73, 74). In CD-associated *NOD2* shift-mutant pure cells, mutant NOD2 fails to recruit ATG16L1 to the plasma membrane, thereby limiting the autophagic response triggered by intracellular bacterial infection (73). The defective autophagic clearance of such NOD2-associated intracellular bacteria, such as *S. typhimurium*, adherent invasive *E. coli*, and *Shigella fowleri*, can be reversed by the pharmacological induction of autophagy with the autophagy activator rapamycin (73–75). In addition, CD4+ T cell-dependent immunity to bacterial antigens requires effective antigen presentation for the activation of NOD2-mediated autophagy in dendritic cells (74).

#### 3.1.4 LRRK2

Initially, gene *LRRK2* mutations were investigated in association with the onset of Parkinson’s. Subsequent studies revealed that functional *LRRK2* variants also increase the genetic risk of CD (35, 76). LRRK2 protein kinase is a complex enzyme with kinase and guanosine triphosphatase activities. *LRRK2* missense mutations cause overactive LRRK2 protein kinase. The inhibition of LRRK2 protein kinase activity was shown to lead to autophagy stimulation in an atypical manner, a pathway that is independent of the mammalian target of rapamycin (mTOR) and ULK1 but dependent on the activation of BECN1-containing class III PI3 kinases (77). *LRRK2*-deficient mice exhibit high susceptibility to experimental colitis through modulation of the innate immune response (78). In contrast, recent studies have shown that upregulation of *LRRK2* expression promotes intestinal inflammatory responses (79–81). Because LRRK2 protein kinase is able to bind to K48 and induce its ubiquitination, and it also induces degradation of the autophagy initiator Beclin-1 and phosphorylation of Beclin-1 (81), which is a Beclin-1 inactivation mechanism (82). However, this only indicates the susceptibility to intestinal inflammation after exposure to inflammatory stress (81).

### 3.2 Crosstalk of multiple phenotypes with autophagy is involved in IBD

#### 3.2.1 Endoplasmic reticulum stress interacts with autophagy and is associated with IBD

Endoplasmic reticulum stress (ERS) is the response that activates signaling pathways such as the unfolded protein response (UPR), endoplasmic reticulum overload response, and the caspase-12-mediated apoptotic pathway. These responses can respond to misfolded and unfolded protein aggregation in the lumen of the endoplasmic reticulum and disturbed calcium ion homeostasis. UPR has been found to be initiated by three ER transmembrane sensors, including inositol-requiring enzyme 1 (IRE1), protein kinase R-like ER kinase (PERK) and activation of transcription factor 6 (ATF6). Some of the mediators released by ERS can directly induce autophagic vesicle formation and initiate autophagy (83). Findings from recent studies have also confirmed that ERS induces autophagy through multiple pathways, such as the UPR pathway and Akt signaling (84). The induction of autophagy and suppression of inflammation occurs through the inhibition of the PI3K/Akt/mTOR pathway (85, 86).

In the course of IBD, on one hand, ERS regulates autophagy. ER degradation is associated with increased ATF6 activity in the colonic mucosa of patients with UC (87). ERS activates autophagy through the ATF6 -mediated upregulation of death-associated protein kinase 1 (DAPK1) and mediates mAtg9 translocation (88). In IECs this process enhances autophagy for killing bacteria (89). ATF6α acts as an intermediate signaling molecule that regulates its upstream signaling factors and downstream molecules XBP1 and Atg16L1. It is involved in the interaction between ERS and IECs autophagy in IBD (90). In addition, UPR and autophagy also intersect in the PERK-EIF2α-ATF4 pathway (87, 91, 92), where ATF4 induces the expression of the pro-apoptotic cytokine CHOP and increases the transcription of the autophagy gene ATG5 (93). It also induces the expression of DNA damage response 1 (REDD1) during ERS (94), and REDD1 expression in intestinal neutrophils activates autophagy by inhibiting mTOR phosphorylation, which is closely related to the severity of UC (95). Finally, ERS stimulates the IRE1/TRAF2/apoptosis signaling regulatory kinase-1/JNK and IRE1/XBP1 signaling axes to release Beclin1 to increase autophagy (96, 97). On the other hand, autophagy regulates ERS, as evidenced by the fact that the impairment of autophagy promotes endoplasmic reticulum stress. The inhibition of trigger receptor-1 expression on myeloid cells reduces ERS in mice with colitis by restoring macroautophagy and CMA (98). In mice with inactivated IKKα kinase, loss of IKKα function resulted in the reduced stability of ATG16L1, which induced UPR and significantly impaired intestinal epithelial regeneration in mice with acute colitis model (99). Similarly, *Atg16L1* knockout mice exhibit more severe colonic inflammation owing to deficient autophagy, resulting in the insufficient degradation of ER to nucleus signaling 1, allowing its excessive accumulation and activation (100).

Appropriate ERS can maintain homeostasis in the intestine, but excess ERS can induce IEC apoptosis and intestinal mucosal barrier dysfunction, and inducing pro-inflammatory cytokine production. Autophagy can also alleviate IBD in several ways; ERS can activate autophagy, but excessive autophagy exacerbates apoptosis and promotes the development of IBD. Therefore, it will be meaningful to explore how the interaction between ERS and autophagy influences the development of IBD, which will help to further explore the pathogenesis of IBD.

#### 3.2.2 Intestinal antimicrobial defense interacts with autophagy and is associated with IBD

Antimicrobial defense is known to be closely related to the expression of autophagy genes (101, 102). Intracellular pathogens attempt to disrupt cell membranes to establish replication niches, cross cell membranes into different compartments, or simply avoid degradation. Autophagy and related pathways can limit such processes to complete the life cycle (103). For extracellular pathogens, autophagy limits infection-induced damage by supporting the activity of specific cell types or inhibiting the production of pro-inflammatory cytokines (103). A network to elucidate the interaction between mucosal bacteria and host autophagic signaling through human intestinal mucosal biopsies showed that patients with CD exhibited greater autophagy and associated signaling cascades than patients with UC. Patients with UC exhibited more severe dysbiosis and a functional phenotype of intestinal mucosal colonizing bacteria (104, 105). Moreover, the populations of the dominant and low-abundance bacteria were positively and negatively correlated with the expression of host autophagy genes, respectively (104). Gene *ATG7* conditional knockout mice of IEC exhibited more abundant bacterial invasion and a significant decline in antibacterial or antiparasitic peptide expression in the colonic epithelium (106). Similarly, *ATG5*-deficient mice showed a decrease in inflammation-controlling bacteria and an increase in the number of pro-inflammatory bacteria (107).

An increased amount of IgA-coated bacteria, which were confirmed as IBD-promoting microbes (108), in the feces of mice with myeloid cell-specific Atg16l1 deficiency and in CD patients carrying the *ATG16L1*T300A variant (109). The interactive effects of altered *ATG16L1* expression on bacteria in IECs and macrophages are widely recognized. Mice with dendritic cell *ATG16L1* deletion showed increased susceptibility to infection in DSS-induced colitis (110) but not to *S. typhimurium* (111). However, *ATG16L1* regulates IECs autophagy and is necessary for the clearance and control of *S. typhimurium* transmission in studies of autophagy gene-specific deletion in IECs (111, 112). Also, CD-associated adherent-invasive *E. coli* persist in autophagy-deficient IECs. However, it has been shown that autophagy deficiency in *ATG16L1* hypomorphic mice alleviates bacterial burden and protects the mouse intestine from severe inflammatory damage (113, 114). Some intestinal bacteria deliver immunomodulatory molecules to immune cells through secretory outer membrane vesicles. *Chu* and his colleagues (115) showed that outer membrane vesicles require expression of the IBD-related genes *ATG16L1* and *NOD2* to activate the atypical autophagic pathway. However only in *Bacteroides fragilis* infection, dendritic cells lacking *ATG16L1* fail to induce regulatory T cells to exert a suppressive effect on mucosal inflammation. This also describes genetically how the interaction between autophagy and microbes synergistically promotes beneficial immune responses.

In addition, autophagy can also affect intestinal antimicrobial defense by influencing the formation of secretory granules. The alternative autophagy-based secretion pathway in the atypical secretion of lysozyme is known as secretory autophagy (116). The disruption of secretory autophagy in mouse Paneth cells results in the increased risk of CD in humans when *ATG16L1* is mutated (116). In summary, autophagy plays an overall positive role in intestinal bacterial defense, but it has a high degree of cell-type specificity and infection-type specificity in their interactions with the intestinal microbiota

Conversely, host cell autophagy was also influenced by the composition of the intestinal microbiota; e.g., fecal microbiota transplantation increased levels of autophagy-related proteins in the intestinal mucosa of piglets (117). Likewise, bacterial metabolites exert a critical effects on energy homeostasis and autophagy in IECs. For example, colonic cells in germ-free mice are in a state of energy deprivation. In this state, colonic IEC autophagy is enhanced, and when butyrate is added to germ-free colonic cells, it compensates for the deficiency in mitochondrial respiration and limits autophagy (118).

#### 3.2.3 Autophagy-regulated apoptosis is associated with intestinal barrier function in IBD

The strict regulation of autophagy and apoptosis plays a critical role in maintaining tissue homeostasis. Autophagy maintains cell survival in the presence of multiple cellular stressors by degrading long-lived proteins and damaged organelles, while apoptosis eliminates damaged and mutated cells. Autophagy has been shown to exert an inhibitory effect on apoptosis in liver disease (119) and neurological disorders (120). *Pott* and his colleagues (48) found that mice with IBD selectively deficient in *ATG16L1* in IECs showed increased TNF-induced apoptosis, which exacerbated intestinal pathology. However myeloid *ATG16L1* deficiency a exerted limited effect on disease. Autophagy protein ATG16L1 in the intestinal epithelium is critical for preventing Paneth cell loss and excessive cell death in an animal model of virus-induced IBD (121). The above findings suggest that the IEC autophagic process can control inflammation-induced apoptosis to maintain intestinal barrier integrity, thereby limiting chronic intestinal inflammation. Alpinetin was shown to enhance intestinal barrier function by driving autophagy to inhibit IEC apoptosis (122). The intracellular protein High Mobility Group Box 1 regulates apoptosis and attenuates inflammation-associated cell injury by protecting the autophagy proteins BECN1 and ATG5 from calpain-mediated cleavage during inflammation (123).

### 3.3 Autophagy-related signaling pathways are associated with intestinal homeostasis in IBD

#### 3.3.1 mTOR

mTOR is an atypical serine/threonine kinase that interacts with specific junction proteins to form two different macromolecular complexes, mTORC1 and mTORC2. mTORC2 primarily controls the cytoskeleton and motility, whereas mTORC1 primarily controls important cellular processes, including autophagy. Autophagy is negatively regulated by upstream mTOR, and the pharmacological effects of mTOR inhibitors and autophagy stimulators significantly improve experimental colitis and oxidative stress *in vivo* (124).

##### 3.3.1.1 AMPK/mTOR signaling pathways

Adenosine 5’-monophosphate (AMP)-activated protein kinase (AMPK), a key molecule in the regulation of biological energy metabolism, is an AMP-dependent protein kinase. Sodium hydrosulfide (NaHS) activates hepatic autophagy through the AMPK/mTOR pathway and improves non-alcoholic fatty liver disease (125). In colonic tissues, NaHS restored impaired cellular autophagy caused by lipopolysaccharide (LPS) stimulation in rats *in vitro* and *in vivo* while improving the signs of UC (126). *Xiong* and his colleagues (127) found that Sinensetin inhibits apoptosis and alleviates intestinal barrier dysfunction in colitis by promoting autophagy of epithelial cells, but this effect could be reversed by AMPK knockdown. Further, data from *Arab* (128) showed that dapagliflozin activates the AMPK/mTOR pathway by increasing pAMPK (Thr172)/total AMPK levels and decreasing the p-mTOR (Ser2448)/total mTOR ratio. This is consistent with the action of metformin (an activator of the autophagy machinery) through an AMPK-dependent signaling pathway for limiting dextran sulfate sodium (DSS)-induced damage to the intestinal barrier (129). Similarly, nicotine ameliorates the severity of DSS-induced colitis in a mouse model of UC by modulating AMPK/mTOR pathway-mediated autophagy and regulating apoptosis and proliferation (130).

##### 3.3.1.2 mTOR/NLRP3 signaling pathways

NOD-like receptor thermal protein domain associated protein 3 (NLRP3), a component of inflammatory vesicles, is responsible for the activation of caspase-1 and subsequent pro-IL-1β and pro-IL-18 maturation. It has long been shown that single-nucleotide polymorphisms in the NLRP3 gene are associated with susceptibility to CD (131). There is a reciprocal regulatory relationship between NLRP3 expression and autophagy, with activation of autophagy limiting NLRP3 activity and thereby moderating inflammation (132).

*Jesus* (133) showed the following: 1) NLRP3 is a novel binding partner for mTOR; 2) NLRP3 plays a key role in the structural blockade of autophagy by promoting mTOR phosphorylation; 3) hypoxia attenuates the colonic inflammatory response by downregulating the binding of mTOR and NLRP3 and activating autophagy. A study by *Mai* et al. demonstrated that palmatine ameliorated intestinal inflammation by reducing mitochondrial reactive oxygen species (mtROS) production and inhibiting NLRP3 inflammatory vesicles through mitochondrial autophagy (134). Thus, promoting mitochondrial autophagy may be a method to terminate the hyperinflammatory response by inactivating NLRP3 inflammatory vesicles. Andrographolide prevents inflammation-associated colon cancer through the mitochondrial autophagy-mediated inhibition of NLRP3 inflammatory vesicles (135). Similarly, the inhibition of NRLP3 inflammatory vesicles may be an effective mechanism to promote autophagy activation to improve colitis. Ginsenosides, which are anti-inflammatory molecules, were reported to inactivate NLRP3 inflammatory vesicles, thereby inducing mitochondrial autophagy to improve DSS-induced experimental colitis (136).

##### 3.3.1.3 AKT/mTOR signaling pathways

AKT, also known as protein kinase B, is a serine/threonine-specific protein kinase that plays a key role in various cell growth processes. The activation of AKT/mTOR signaling pathway negatively regulates autophagy, and AKT/mTOR signaling participates in pathogenesis of IBD through the regulation of autophagy (137, 138). For example, heat shock transcription factor 2, which promotes autophagy in IECs by inhibiting the PI3K/AKT/mTOR signaling pathway, plays a protective role in UC (139). The overexpression of ring finger protein 8 reduces AKT and mTOR phosphorylation, increases autophagy, and improves intestinal inflammation in UC mice (140). Xianglian pill was also shown to promotes cellular autophagy and attenuates DSS-induced acute colitis in mice by blocking the activation of the PI3K/Akt/mTOR signaling pathway (141).

#### 3.3.2 NF-κB signaling pathways

NF-κB is a transcription factor that activates the transcription of pro-inflammatory factor genes and plays a critical role in the cellular inflammatory response and immune response. The atypical stimulation of NF-κB upregulates of the expression of two activators of selective autophagy, BAG3 and HspB8, thereby regulating the autophagic process (142). Neural-precursor-cell-expressed developmentally down-regulated 4 is a ubiquitin ligase that regulates Beclin-1 stability. NF-κB causes Beclin-1 division to inhibit autophagy through the upregulation of Neural-precursor-cell-expressed developmentally down-regulated 4 (143). Similarly, autophagy inhibits NF-κB activation by inhibiting the NF-κB upstream regulator IκB kinase (IKK) (144) or by recruiting phosphorylated IKK to the autophagic vesicle compartment (145). In addition, ATG16L1 negatively regulates the pro-inflammatory cytokine response through the downregulation of NF-κB expression (146). Autophagy deficiency owing to ATG16L1^T300A^ polymorphism in macrophages promotes the NF-κB-dependent cytokine response and puts CD at an increased risk of disease (147). Ginseng polysaccharides exert beneficial effects, such as regulating intestinal microbiota, protecting the intestinal mucosal barrier, and promoting autophagy. Toll-like receptors (TLR) are key receptors for pathogen recognition and immune activation and are widely present in various immune cells and epithelial cells. Ginseng polysaccharides were demonstrated to improve DSS-induced intestinal inflammation by inhibiting the TLR4-NF-kB pathway and activating mTOR-dependent autophagy (148).

#### 3.3.3 Nrf2/OH-1 signaling pathways

Nuclear transcription factor E2-related factor 2 (Nrf2) is a key transcription factor in the cellular antioxidant response. The pathway involving heme oxygenase-1 (HO-1) is a key signaling pathway that regulates endogenous oxidative stress. Reportedly, p62 SQSTM1 is a target gene of Nrf2 (149), and the Keap1/Nrf2 pathway is strongly associated with autophagy through the autophagic adapter p62 SQSTM1 protein (150). The activation of the Nrf2/HO-1 pathway effectively controlled colonic tissue damage in a trinitro-benzene-sulfonic acid (TNBS)- and DSS-induced colitis model (151, 152). *Arab* (128) showed that dapagliflozin activates autophagy at least in part by activating the Nrf2/OH-1 pathway to reduce the severity of drug-induced colitis in rats.

#### 3.3.4 Other signaling pathways

The hypoxia-inducible factor-l-dependent induction of Wnt1 disrupts autophagy in epithelial cells, suggesting a role of the Wnt signaling pathway in impaired autophagy in the mucosal epithelial cells of patients with IBD (153). Signal sensors and activator of transcription 3 (STAT3) is a key molecular pathway that can be activated by various pathogens and participates in complex immune disorders (154, 155). *Zhang* (156) showed that the deletion of High Mobility Group Box 1 protein in the intestine leads to the abnormal regulation of STAT3, which inhibits autophagy. The author also showed that the inhibition of STAT3 restores the autophagic response in bacteria-infected cells, suggesting that STAT3 activation should occur upstream of autophagy regulation. In *in vivo* and *in vitro* models of neonatal necrotizing small bowel colitis, TNF-α may induce autophagy through the extracellular signal regulated kinase 1/2 pathway, regulate the apoptosis of neonatal necrotizing small bowel colitis cells IEC-6, and promote disease progression (157). In *ATG5*-silenced IECs, the MAPK/ERK kinase pathway is activated, which is associated with elevated levels of inflammatory cytokines (158).

## 4 The role of autophagy in CRC

IBD is at risk of developing into CRC. Based on the key role of autophagy in IBD, the role of autophagy in CRC has also gained attention in recent studies. Autophagy is now emerging as a potent regulator of tumorigenesis by protecting cells from metabolic stress and oxidative damage. However, recent data show that autophagy may promote tumour growth and progression in some conditions (159, 160). Thus, the role of autophagy in tumorigenesis is equally complex.

The role of *UVRAG* as a tumor suppressor gene has been described among other autophagy-related genes (161). *UVRAG* regulates BECN1 expression, suggesting that the interaction between UVRAG and BECN1 may be a condition for the tumor suppressive effect. In colon cancer cell lines carrying *UVRAG* (c.709delA) deletion, reduced UVRAG levels were observed to correlate with impaired autophagy induction (161, 162). Nonsynonymous single-nucleotide polymorphism in *ATG16L1* ^T300A^ was associated with improved overall survival and increased basal production of type I interferon in human CRC (163). In addition, a study analyzing gene *ATG5* mutation and expression in gastrointestinal tumors showed that ATG5 protein was well expressed in normal colonic mucosal epithelial cells but was absent in 23% of CRC (164).

In addition to its direct anti-tumor effects, autophagy inhibits CRC by weakening the inflammatory response in the tumor microenvironment and increase the processing and presentation of tumor-associated antigens. This improves anti-tumor immunity. Some chemotherapeutic agents with immunogenic properties have been shown to exhibit immunogenic anti-tumor properties by inducing autophagic cell death (165). Bacillus Calmette - Guerin has been shown to induce autophagic cell death through TLR2 and TLR4 signaling and to radiosensitize colorectal cell lines. *In vivo* experiments have further demonstrated that Bacillus Calmette - Guerin -mediated radiosensitization is an autophagy-dependent phenomenon (166). In contrast, preoperative hydroxychloroquine treatment improved the therapeutic response to 5-fluorouracil and radiotherapy in patients with advanced CRC. However, chloroquine can inhibit autophagy by blocking the fusion of autophagosomes and lysosomes, sensitizing HT-29 CRC cells to chemotherapy and irradiation (167). In addition, when autophagy genes (e.g., *ATG5* or *BECN1*) were knocked out, cell death and ROS production were enhanced by the oxaliplatin treatment of Caco-2 cells (168, 169). Thus, during CRC, autophagy can both promote tumor survival and lead to tumor cell death, depending on tumor type, CRC stage, and metabolic environment.

## 5 Pharmacological studies on autophagy in the treatment of IBD

In terms of drug research, the therapeutic targets of IBD are primarily elements related to the maintenance of homeostasis in the intestine, and autophagy has emerged as a new potential therapeutic option for it. *Wang* and his collaborators (170) then assessed the therapeutic effects of 37 Food And Drug Administration -approved autophagy activators using an embryonic anthracycline-induced cardiotoxicity zebrafish model. They identified spironolactone, pravastatin, and minoxidil as top-ranking drugs for reversing the decline in cardiac function in anthracycline-induced cardiotoxicity and proved that spironolactone and rapamycin activated autophagy in an ATG7-dependent fashion (170). In intestinal diseases, glutamine has been widely used for damage repair in the intestinal mucosa, and reportedly, glutamine can promote IEC autophagy through the mTOR and p38 MAPK signaling pathways, thereby inhibiting stress-induced apoptosis (171). Anakinra, a human IL-1 receptor antagonist produced by genetic recombination technology, has been used in rheumatoid arthritis. Studies have demonstrated that anakinra can restore autophagy levels and reduce il-1-mediated inflammatory responses in patients with chronic granulomatosis, thereby protecting against TNBS-induced colitis, reducing the severity of chronic granulomatosis and promoting recovery from rectal abscesses (15). Sirolimus and everolimus are two rapamycin analogs that have been used clinically. In a retrospective study of refractory IBD that did not respond to conventional treatment in children, 45% of patients with UC and 100% of patients with CD showed clinical remission with sirolimus (172). Meanwhile, treatment with everolimus (an autophagy inducer) also improved CD-like ileitis in IL-10-deficient mice (173). However, whether the rapamycin analogs in the above cases act through autophagy and possibly immunosuppression remains unclear. Furthermore, it is interesting to note that epidemiological studies have shown that smoking is beneficial in UC (174) that the ameliorative effect of nicotine on intestinal inflammation may contribute to this (175–177). *Gao* and his colleagues (130) confirmed that nicotine ameliorates the severity of DSS-induced colitis in a mouse model of UC by regulating AMPK/mTOR pathway-mediated autophagy and inhibiting apoptosis and proliferation.

However, few of the current abundant of activators or inhibitors have strictly selected one autophagic pathway and one target. Therefore, some of the research has turned towards identifying small m *Chu* and his colleagues olecules and peptides to precisely modulate autophagy in pathological processes. For example, P140 is a peptide that selectively targets the autophagic process, particularly CMA. P140 effectively affects the processing of endogenous antigens as well as downstream deleterious pro-inflammatory events by interfering with the over-activated autophagic process (178). Importantly, the normal immune system is not affected by this (179) and P140 has been shown to be safe in clinical trials involving patients with systemic lupus erythematosus (180, 181). Preliminary data from current studies also favor P140 as a valuable tool for the treatment of IBDs (182). Spermidine is a natural polyamine that has been shown to improve the weight loss and colonic damage seen in mice with DSS-induced IBD (183). Liu’s study suggests that the improvement of IBD by spermidine is partly due to its activation of macrophage autophagy, allowing it to acquire anti-inflammatory properties (184).

Similarly, some herbal extracts or active ingredients have been shown to ameliorate intestinal inflammation by inducing autophagy; e.g., andrographolide inhibits NLRP3 inflammatory vesicles by enhancing autophagy (135), and tretinoin enhances autophagy by inhibiting the PI3K/AKT/mTOR signaling pathway (138).

Autophagy, owing to its fundamental role in maintaining homeostasis in the intestine, can be an attractive therapeutic target. Although some progress has been made in the study of drugs that induce autophagy through different mechanisms, the treatment of IBD by autophagy modulation is still in its preliminary stages. It is challenging because of the low pharmacological specificity of its targets, the lack of specificity for specific cell classes, and the autophagy-independent effects. Therefore, extensive experimental studies and large-scale clinical trials are necessary to explore and confirm the results.

## 6 Discussion

Autophagy affects the intestine *via* multi-pathway and cell-specific and environment-specific mechanisms. The microbial-autophagy-IBD interaction has been described previously, but the diet-autophagy-IBD interaction is poorly understood. Currently, the effects of vitamin D in diet-autophagy-IBD interaction have been studied. The deficiency of this vitamin has been recognized as an important pathological basis for IBD (185). The vitamin D receptor enhances autophagy through Beclin-1 (186), reduces necroptosis (187), and promotes the release of the tight junction protein Claudin-2 to slow intestinal inflammation (188). In addition, anthocyanins belong to a subclass of dietary flavonoids (polyphenolic compounds) that are important in preventing the onset and progression of intestinal inflammation (189) and can accelerate autophagy by regulating the PI3K/AKT/mTOR signaling pathway (190). *Haihua* and his colleagues further demonstrated that proanthocyanidins may alleviate DSS-induced UC by inducing autophagy (191). Therefore, there are many gaps in our understanding of the diet-autophagy-IBD relationship. These gaps need to be filled with evidence from more experimental and clinical studies.

To date, the number of studies on microautophagy and CMA is insufficient in IBD, and it is yet to be confirmed whether autophagy governs the age and gender in IBD onset. In genetics, the development and application of genetic testing will be particularly important in predicting the occurrence, type, and prognosis of IBD as well as the guidance and utility of drugs, especially for patients with refractory IBD. However, these aspects are still in the preliminary stage of research.

Overall, the current findings indicate that the absence of autophagy favors the exacerbation rather than the induction of intestinal inflammation. However, the inhibition of autophagy can ameliorate intestinal inflammation under certain conditions, such as after appendectomy (192). Therefore, a generalized review of the specific pathways involved in autophagic mechanisms of action in IBD is necessary to further explore the interactions between the environment, infection, and genetics. This will help us to understand how and by what mechanisms autophagy affects the gut and interacts with other cellular processes under specific conditions. These findings may be useful in developing individualized therapeutic regimens.

## 7 Conclusion

**Figure 2:**
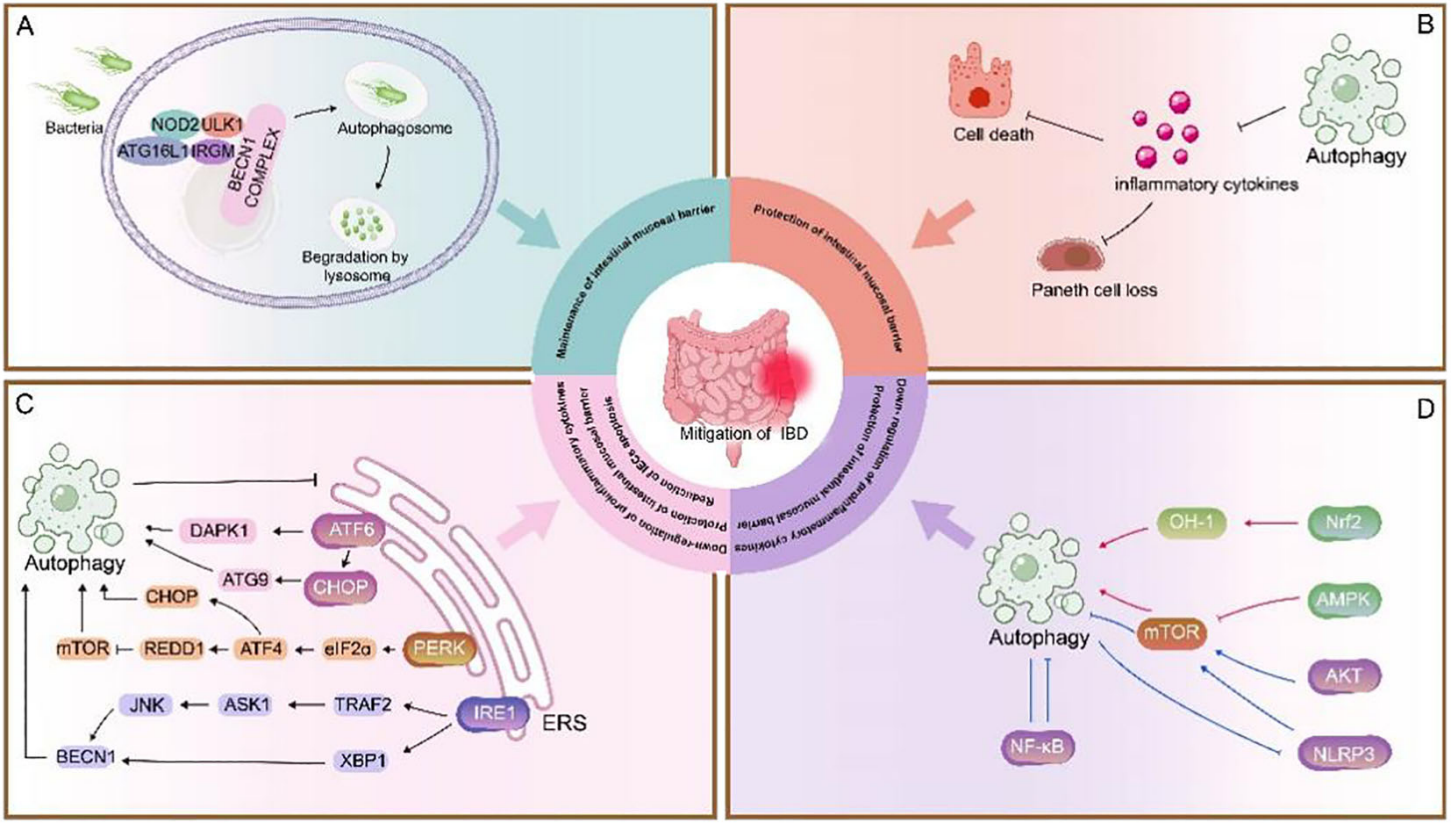
Role of autophagy in IBD. **(A)** NOD2 recruits ATG16L1 to the plasma membrane at the bacterial entry site, initiating autophagy. Association of IRGM with NOD2 promotes IRGM ubiquitination and the assembly of the core autophagy machinery, promoting xenophagy and intracellular bacterial clearance (135). This process is important for maintenance of intestinal mucosal barrier (48, 121). **(B)** Autophagy inhibits the secretion of inflammatory cytokines to prevent loss of Paneth cells and exaggerated cell death. **(C)** The UPR is activated in response to ERS and consists of three pathways: ATF6, PERK, and IRE1. These three stress-related proteins bind to GRP78 when the cell is in a steady state. Under ERS, GRP78 separates from these three receptors and activates the ATF6, PERK, and IRE1 pathways. 1) ATF6 is cleaved in the Golgi apparatus, binds to specific DNA, regulates the pro-apoptotic cytokine CHOP, mediates mAtg9 transport, and activates autophagy; it also activates autophagy by upregulating DAPK1 (88, 89). 2) PERK activates eIF2α through autophosphorylation, thereby activating ATF4, which induces the expression of CHOP and the upregulation of autophagy genes. ATF4 also induces the expression of REDD1 and inhibits mTOR phosphorylation to activate autophagy (87, 91–93). 3) IRE1 splices XBP1 into its active form and can bind to TRAF2 to activate JNK, thereby upregulating Beclin-1 and promoting autophagy. Autophagy and ERS are mutually regulated (96, 97). ERS can promote autophagy, and when ERS is overactive, activated autophagy can inhibit ERS, thereby reduction of IECs apoptosis, down-regulation of proinflammatory cytokines, protection of intestinal mucosal barrier. **(D)** In this section, the red line represents the Pro-autophagy pathway pathway and the blue line represents the inhibition of autophagy pathway. Autophagy is primarily regulated by the following pathways:1) mTOR is a key negative regulator of autophagy. The AMPK/mTOR pathway positively regulates autophagy (125); The NLRP3/mTOR and AKT/mTOR pathways negatively regulate autophagy (133, 137), whereas the upregulation of autophagy also inhibits NLRP3 inflammatory vesicle activation (132). 2) Nrf2-OH pathway positively regulates autophagy (150).3) The NF-κB pathway negatively regulates autophagy and vice versa (142, 144, 145). Autophagy-related signaling pathways benefit protection of intestinal mucosal barrier and down-regulation of proinflammatory cytokines, protection.

Great strides have been made in our understanding of autophagy within intestinal cells both in a healthy and diseased context. A growing number of autophagic genes and proteins have been shown to be closely associated with intestinal inflammatory processes. Autophagy genes or proteins reduce susceptibility to intestinal injury and maintain intestinal homeostasis by providing endoplasmic reticulum stress protection, phagocytosing pathogen invasion, reducing pathogen attachment, and regulating apoptosis (**Figure 2**). In the study of autophagy-related signaling pathways and IBD, autophagy protects the intestinal mucosal barrier mainly by regulating cytokines and modulating apoptosis (**Figure 2**). In addition, as the anti-tumor effects of some chemotherapeutic drugs have been found to be associated with autophagy, the application of autophagy in cancer has received increasing attention. A better understanding of the molecular mechanisms of crosstalk between apoptosis and autophagy may be the key in identifying novel applications of combinatorial treatment to CRC. In pharmacological studies, although some autophagy inducers applied in the clinic have been found to be effective in certain diseases, the studies are still at preclinical stage, and more evidence is needed based on clinical tests.

Autophagy is highly cell-specific and environment-specific. Therefore, strict selection of autophagic pathways or targets without affecting other biological processes is the key to autophagy-based therapeutic strategies for IBD. In the future, additional efforts are needed to validate the suitability of autophagy as a therapeutic target for IBD and to determine whether it is effective and feasible. These efforts should perhaps be devoted to the identification of small molecules or peptides for the precise modulation of the autophagic process and to gradually transfer experimental findings to clinical studies.

## Author contributions

S-LC, C-ML, WL, Q-SL, D-SH and SY-H retrieved and analyzed concerned literatures. S-LC and Q-SL wrote the manuscript. S-LC, C-ML, WL, M-YZ, J-HZ, S-YH, Y-WH, and YZ revised the manuscript.All authors contributed to the article and approved the submitted version.
